# Genetic diversity, population structure, and genome-wide association analysis of ginkgo cultivars

**DOI:** 10.1093/hr/uhad136

**Published:** 2023-07-11

**Authors:** Yaping Hu, Zhaoyan Yu, Xiaoge Gao, Ganping Liu, Yun Zhang, Petr Šmarda, Qirong Guo

**Affiliations:** Co-Innovation Center for Sustainable Forestry in Southern China, Nanjing Forestry University, Nanjing 210037, China; Coconut Research Institute of Chinese Academy of Tropical Agricultural Science, Wenchang, Hainan 571339, China; Co-Innovation Center for Sustainable Forestry in Southern China, Nanjing Forestry University, Nanjing 210037, China; Co-Innovation Center for Sustainable Forestry in Southern China, Nanjing Forestry University, Nanjing 210037, China; Institute of Grassland, Flowers, and Ecology, Beijing Academy of Agriculture and Forestry Sciences, Beijing 100097, China; Department of Botany and Zoology, Faculty of Science, Masaryk University, Koltlářská 2, Brno 61137, Czech Republic; Co-Innovation Center for Sustainable Forestry in Southern China, Nanjing Forestry University, Nanjing 210037, China

## Abstract

*Ginkgo biloba* is an economically valuable tree worldwide. The species has nearly become extinct during the Quaternary, which has likely resulted in reduction of its genetic variability. The genetic variability is now conserved in few natural populations in China and a number of cultivars that are, however, derived from a few ancient trees, helping the species survive in China through medieval times. Despite the recent interest in ginkgo, however, detailed knowledge of its genetic diversity, conserved in cultivated trees and cultivars, has remained poor. This limits efficient conservation of its diversity as well as efficient use of the existing germplasm resources. Here we performed genotyping-by-sequencing (GBS) on 102 cultivated germplasms of ginkgo collected to explore their genetic structure, kinship, and inbreeding prediction. For the first time in ginkgo, a genome-wide association analysis study (GWAS) was used to attempt gene mapping of seed traits. The results showed that most of the germplasms did not show any obvious genetic relationship. The size of the ginkgo germplasm population expanded significantly around 1500 years ago during the Sui and Tang dynasties. Classification of seed cultivars based on a phylogenetic perspective does not support the current classification criteria based on phenotype. Twenty-four candidate genes were localized after performing GWAS on the seed traits. Overall, this study reveals the genetic basis of ginkgo seed traits and provides insights into its cultivation history. These findings will facilitate the conservation and utilization of the domesticated germplasms of this living fossil plant.

## Introduction

*Ginkgo biloba* L. is a world-renowned living fossil plant and the last extant representative of a large group of Mesozoic gymnosperms. Ginkgo was widespread in the Northern Hemisphere, based on available fossil evidence, and became nearly extinct with the climatic oscillations of the Quaternary [1, 2]. Today, ginkgo survives only in three refugia in eastern, southwestern, and southern China [3] and is extremely rare, being still included in the International Union for Conservation of Nature (IUCN) red list of endangered species [4–6]. Beyond the natural populations, its survival through the medieval times was also supported by its popularity and cultivation by Buddhist monks [7]. Material from these remaining ginkgo resources has been distributed in modern times to all continents [8, 9] and ginkgo is now cultivated in large quantities, especially in China, particularly for landscaping, wood, food processing, and extraction of medicinal ingredients [10].

While the genetic diversity of ancient populations of ginkgo is relatively well known, it remains largely unknown what genetic variation may be hidden in ancient trees and a number of cultivars derived from the various existing ginkgo resources [3, 11, 12]. This absence of knowledge, together with unknown genetic relationships among the existing cultivars, causes difficulties for the efficient conservation of ginkgo genetic resources and the development of germplasm resources. According to the different cultivation uses, a large number of elite cultivars have been selected and bred in the past, such as those with high flavonoid content, high wood volume, large fruit size, etc. Seed cultivars in China are traditionally classified according to the shape index of the seed, i.e. the length of the seed with the outer testa removed divided by its width. Cultivars with elongated seeds and seed shape index ≥1.75 are classified as ‘Changzi’ cultivars, those with 1.50 ≤ seed shape index < 1.75 as ‘Fozhi’ cultivars, those with 1.30 ≤ seed shape index < 1.50 as ‘Zhongzi’ cultivars, and those with rounded seeds with seed shape index <1.30 as ‘Yuanzi’ cultivars. However, there is currently no molecular basis for this classification, and to what extent this classification indeed reflects the genetic variability and relationships among the existing cultivars remains unclear.

Genotyping-by-sequencing (GBS) is a common, simplified genome sequencing technique that is widely used in population genetics [13], and is becoming increasingly popular together with other high-throughput genotyping techniques due to their economic feasibility [14–17]. This method is applied to the study of genetic diversity, domestication history, and key trait-regulatory loci of cultivated populations, e.g. to explore the origin and spread of tomato germplasm [18], the history of artificial domestication of cultivated apples [19], or the identification of *Plasmopara viticola* resistance loci in grapes [20]. Beyond GBS, the high-precision reference genome of ginkgo [21] has made it possible to conduct genome-wide association studies (GWAS) in this species to further explore the associations between phenotypes and markers, single-nucleotide polymorphisms (SNPs), and genes [21].

Here we aimed to study genetic structure, kinship, inbreeding, and average linkage disequilibrium (LD) decay in cultivated ginkgo germplasm, through GBS-based identification of SNPs and runs of homozygosity (ROHs). In addition, we mapped SNPs and candidate genes related to seed length, width thickness, and embryo-free rate based on GWAS. The findings of this study will parse the genetic relationships between the widely cultivated *G. biloba* cultivars and reveal genetic resources underlying the variation of seed traits, which will facilitate efficient conservation and utilization of ginkgo germplasms.

**Figure 1:**
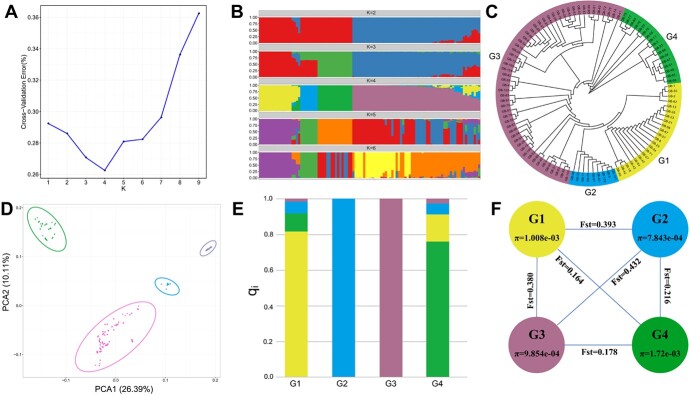
Genetic structure of ginkgo cultivars. **A** Plot of Δ*K* values for *K* from 1 to 9 in STRUCTURE analysis. **B** ADMIXTURE analysis results for *K* = 4–6. **C** Neighbor-joining tree for 102 ginkgo cultivars from 1000 bootstrap replicates. **D** PCA of the 102 ginkgo cultivars. **E** A stacked bar chart showing the pedigrees of different subpopulations (G1–G4) based on the *q*_i_ coefficients derived from the ADMIXTURE analysis. **F** Genetic differentiation index among different subpopulations.

## Results

### Sequencing and SNP calling

The average of 102 samples each yielded 30 227 882 bp of clean data after filtering, and the average effective restriction fragment length was 237 bp. The alignment ratio of clean reads and reference genomes for all samples ranged from 98.15 to 99.34%, with an average of 99.14%.

After alignment and filtering, we identified a total of 9 147 579 variants, of which 8 796 251 were SNPs and the rest were indels, with an average density of one variant/1.12 kb. The overall distribution of variants on each chromosome was relatively uniform (Supplementary Data File S1). Around 90% of the identified variants (8 237 491 out of 9 147 579) were found in intronic regions. In addition, a total of 812 322 variant sites were located in the intergenic region, affecting ~97.35% of all genes in the latest reference genome of ginkgo. The genotypic call rates of all samples met the requirements for subsequent analysis.

**Figure 2:**
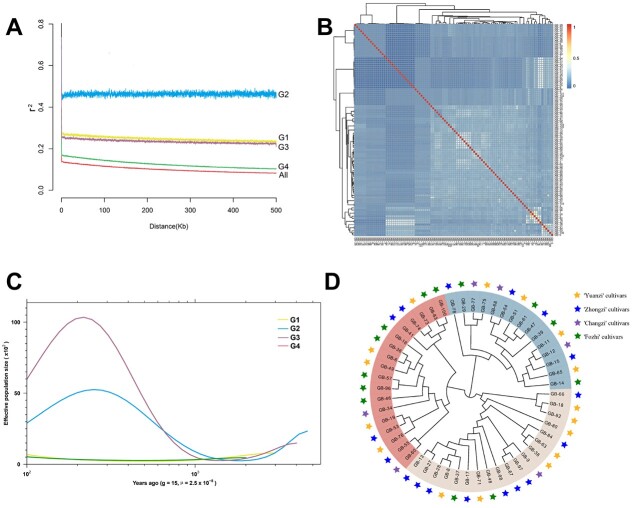
Genetic relationship analysis of ginkgo cultivars. **A** Average LD decay patterns of ginkgo subpopulations. **B** Heat map of paired identity by descent (IBD) values generated between ginkgo cultivars. The phylogenetic tree was estimated based on UPGMA clustering algorithm information. **C** Estimation of historical effective population sizes of different subpopulations based on SMC++. **D** Neighbor-joining tree for female cultivars. The stars on the outermost circle indicate the seed shape index classification.

### Population structure and variation characteristics

The most likely subpopulation classification for all ginkgo cultivars was *K* = 4 after running STRUCTURE starting with *K* = 1 (Fig. 1A and B). We divided all 102 cultivars into four subpopulations, named G1 to G4. Subpopulations G1 and G2 contained 22 and 9 cultivars, respectively, mainly from Tai’an and its surrounding areas in Shandong Province. G3 had the largest number of cultivars, with a total of 54, from different regions of China, including cultivars from Japan and Europe. The remaining 17 cultivars made up the G4 subpopulation, and their geographical origin is likewise relatively dispersed. We constructed a phylogenetic tree of ginkgo cultivars based on high-quality SNPs, which resulted in the same division into four subpopulations (Fig. 1C). The cultivars from Japan and Europe did not show a specific phylogenetic pattern, and were mixed with the cultivars from China. A non-parametric study of genetic structure by principal component analysis (PCA) provided support for the above genetic structure results (Fig. 1D). The first two PCs explain ~36.50% of the variance, with PC1 and PC2 explaining 26.39 and 10.11%, respectively. Subpopulations G1 and G4 showed pedigree contributions from all subpopulations, while subpopulations G2 and G3 showed pedigrees recorded as pure (Fig. 1E). The ancestry of both the G2 and the G3 subpopulation had ~20% contribution from other subpopulations. The G2 subpopulation cultivars mainly originated from the Tai'an area of Shandong Province, China.

The nucleotide diversity (*\ensuremath{\pi}*) of the entire population was 0.001951. The nucleotide diversity of the G2 and G3 subpopulations was significantly lower than that of G1 and G4, showing lower genetic diversity (Fig. 1F). Comparing *F*_ST_ among the four subpopulations, we found that the genetic differentiation index was largest between the G2 and G3 subpopulations, indicating the greatest differentiation between them, while the genetic differentiation between G1 and G4 and between G3 and G4 was smaller. It is generally believed that *F*_ST_ > 0.25 indicates that there is great genetic differentiation among populations.

### Average linkage disequilibrium decay of different subpopulations

LD decay refers to the evolutionary process from linkage disequilibrium to linkage equilibrium between loci. The rate of LD decay tends to differ dramatically among species or among different subpopulations of the same species. The LD decay rate of the G2 subpopulation was much lower than that of the other three subpopulations, and the decay rates of G1 and G3 remained almost the same (Fig. 2A). The LD decay rate of G4 was the fastest among the four subpopulations, slightly slower than that of the whole population. Overall, the LD decay rate of ginkgo is relatively slow, far slower than that of annual crops such as rice [22] and soybean [23], and slower than that of higher-generation perennial woody species such as pear [24] and apple [25]. This may be related to ginkgo’s long lifespan and highly complex super-genome.

### Kin relationships of cultivars

Ginkgo is a dioecious wind-pollinated species with a very wide range of pollen dispersal. The juvenile period is >10 years long, and morphological similarity is difficult to distinguish, which brings great inconvenience to germplasm evaluation and genetic improvement of ginkgo varieties [7]. The absence of obvious affinities among most ginkgo cultivars was shown by the generation of heat maps using the unweighted pair group method with arithmetic mean (UPGMA) hierarchical clustering algorithm (Fig. 2B). However, there was a high probability of genetic relationship between GB-1 and GB-50 and between GB-31 and GB-84.

### Estimation of inbreeding of ginkgo cultivars

**Figure 3:**
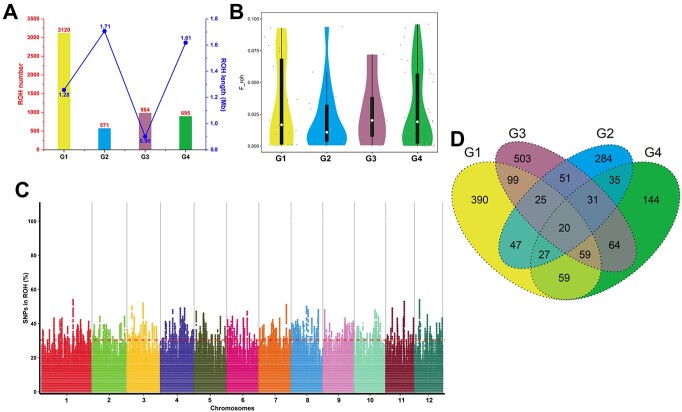
ROH analysis of ginkgo cultivars. **A** Number and mean length statistics of ROHs for each subpopulation. **B***F*_ROH_ distribution of different subpopulations of violins. **C** Frequency distribution of SNPs on chromosomes. **D** Shared genes of ROHs.

Evaluation of inbreeding based on ROHs is a method of evaluating inbreeding using genome-wide information. It can accurately calculate the inbreeding coefficient, and previous studies have proved that the pedigree inbreeding coefficient is lower than the true inbreeding coefficient [26]. The G1 subpopulation had the most ROHs, 3120, which was 670 more than the other three subpopulations combined (Fig. 3A). The G2 subpopulation had the lowest number (571) of ROHs and the longest average ROH length (1.71 Mb). Short ROHs are produced due to inbreeding in more distant generations, and conversely long ROHs are produced due to inbreeding in more recent generations [27]. This indicated that the inbreeding activity of the G3 subpopulation occurred latest. The *F*_ROH_ values of G1, G2, G3, and G4 were 0.022, 0.033, 0.028, and 0.029, respectively (Fig. 3B).

The distribution of ROHs in chromosomes and between chromosomes is affected by many factors, and ROHs will be concentrated in a certain region on the chromosome, called the ROH island. ROH islands are not randomly distributed throughout the genome, but are shared by all individuals in a population. The ROH ratio where the top 1% lies was taken as the threshold line for high-frequency SNPs, and the ROH island was obtained according to the distribution of SNP loci above the threshold in the genome (Fig. 3C). Subpopulations G1, G2, G3, and G4 were found to have 573, 427, 673, and 432 ROH islands, respectively. Subpopulations G1, G2, G3, and G4 were annotated to 390, 284, 503, and 144 specific genes, respectively, after alignment of the ROH island with the reference genome (Fig. 3D). A total of 20 genes were shared by four subpopulations.

**Figure 4:**
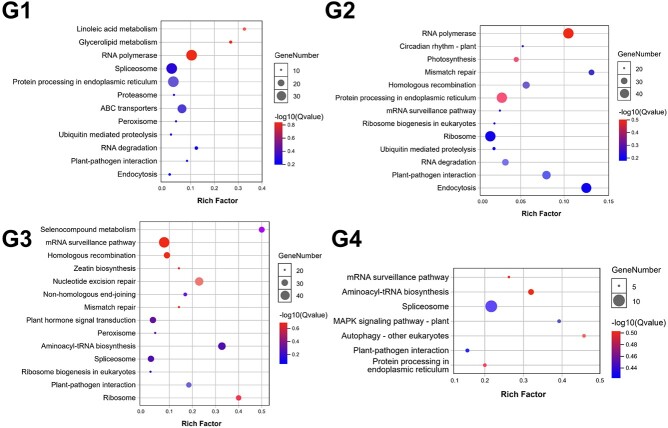
KEGG enrichment analysis of four subpopulations of ROH shared unique genes.

The enrichment functions of genes specific to the four subpopulations were not identical (Fig. 4). The specific genes of the G1 subpopulation were significantly enriched in the two pathways of linoleic acid metabolism and glycerolipid metabolism. Genes specific to the G2 subpopulation were significantly enriched in the photosynthesis and endocytosis metabolism pathways. The same G3 subpopulation-specific genes were mainly enriched in the selenocompound metabolism and ribosome pathways. The number of genes specific to the G4 subpopulation was low compared with the other three subpopulations and was significantly enriched in the autophagy–other eukaryotes and MAPK signaling pathway–plant pathways. The MAPK signaling pathway–plant is extensively involved in plant immune and environmental stress responses in ginkgo [28].

### Population history dynamics based on SMC++

The accuracy of SMC++ analysis for recent population events (within 10 000 years of history) is greatly improved compared with other analysis methods [29]. We chose SMC++ to infer the dynamic historical changes of different subpopulations due to the cultivars being more influenced by human activities. No significant changes in the sizes of the four subpopulations were found until 1500 years ago, with an average of 15 years as a generational cycle (Fig. 2C). About 1500 years ago, the G2 and G3 subpopulations began to expand significantly in size. Around 1500 years ago, during the Sui and Tang dynasties in China, the economy flourished, and the population experienced a significant surge. The practice of cultivating ginkgo, which has been deeply rooted in Chinese culture since ancient times, combined with the prevalent social activities of the era, undoubtedly played a pivotal role in this demographic expansion.

### Seed traits and genome-wide association study

**Figure 5:**
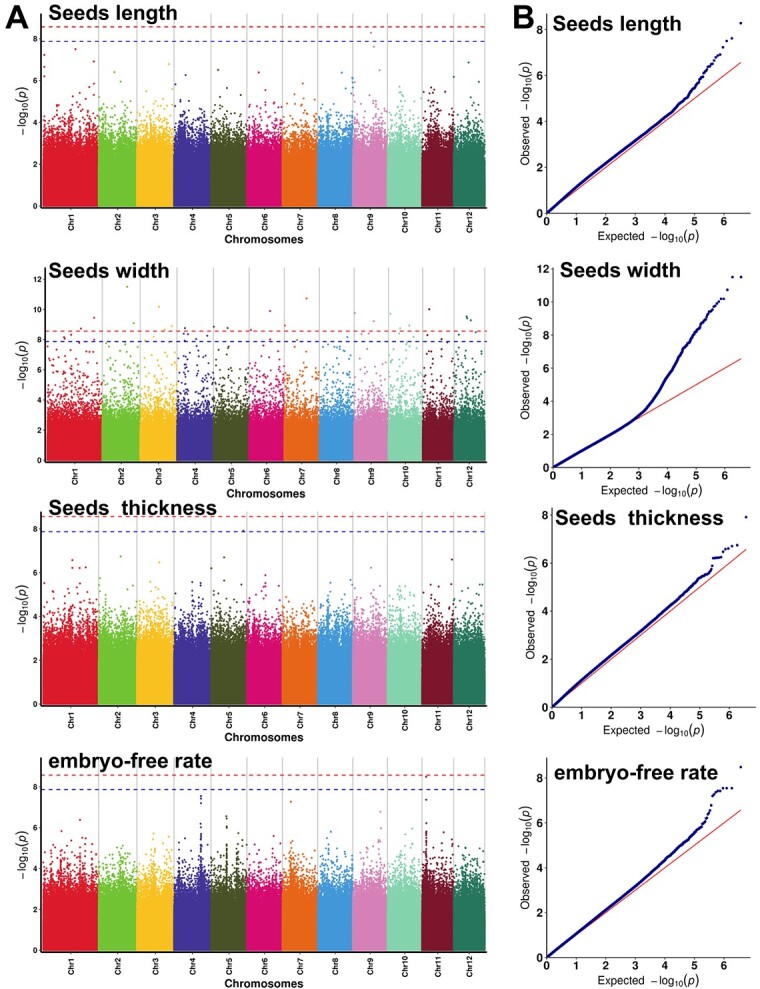
GWAS for seed length, seed width, seed thickness, and embryo-free rate. The upper and lower dashed lines represent associations with false discovery rate corrected p-values less than 0.05 and less than 0.01, respectively. **A** Manhattan plots. **B***Q*–*Q* plots relative to the regression models.

Gene regulation of seeds traits is one of the aspects that concern us most. High-density SNP markers combined with GWAS offer the possibility of identifying quantitative trait loci (QTL) or candidate genes for plant traits. We collected historical data on seed length, width, thickness, and embryo-free rate for 52 cultivars in the China Ginkgo Germplasm Resource Nursery, and used GWAS analysis in ginkgo for the first time. All data conformed to a normal distribution, and the distribution of best linear unbiased predictors (BLUPs) is shown in Supplementary Data File S2. A total of 3 701 576 SNPs were used for GWAS after quality control. A total of 54 SNPs related to ginkgo seed traits were screened and 24 candidate genes were identified (Fig. 5, Table 1).

For seed length, a significantly associated SNP was included in the interval 370.54 to 372.15 Mb on chromosome 9. *Gb_39567* was obtained after gene annotation of this SNP, which was enriched in the transferase activity pathway.

For seed width, a total of 50 significantly associated SNPs were identified, with distribution on all 12 chromosomes. Twenty-two genes were obtained after annotation of all associated SNPs, 10 of which were distributed in the region from 344.23 to 693.35 Mb of chromosome 1. These candidate genes are mainly involved in transcription factors encoding the plant hormones abscisic acid, auxin and ethylene.

For seed thickness, one SNP in the region from 664.01 to 664.93 Mb of chromosome 5 was significantly associated with it. *Gb_20425* was annotated as a candidate gene for regulating seed thickness.

For seed embryo-free rate, only one SNP in the region of 844.28 to 845.64 Mb on chromosome 11 was related to it, and it was annotated to a gene named *CAT* (*Gb_02867*).

*CAT* is a candidate regulator gene for both seed width and embryo-free rate. No SNPs and candidate genes associated with seed weight were found.

### Population structure of ginkgo seed cultivars

The selection and breeding of seed cultivars is one of the important directions for the development and utilization of ginkgo germplasm resources. We used the regions associated with seed traits in the GWAS results to construct phylogenetic relationships for female ginkgo populations. All female cultivars of ginkgo were classified into three major subpopulations according to the phylogenetic structure (Fig. 2D). The phylogenetic classification results and the criteria for classification based on seed shape index mismatch completely. A phylogenetic perspective therefore does not reflect classification based on seed shape index.

## Discussion

The genetic diversity of cultivated populations used to be reduced compared with that of wild populations, which is in line with natural laws [30]. The frequent long-term artificial selection of cultivars has led to a gradual decrease in genetic diversity, making ginkgo face the plight of a huge number of cultivated but endangered species. Compared with cultivated populations, wild populations have higher variability, e.g. ginkgoes of different ploidies were found in wild populations [31, 32]. It is too early to say that the ginkgo has entered the ‘end of evolution’ [33], simply by its long unchanged appearance. We therefore suggest that the collection and conservation of wild populations of ginkgo should be continuously increased, rather than being satisfied with the current level of collection.

The G2 subpopulation basically originated from the Tai’an area of Shandong Province, indicating that rarely large-scale gene flow occurred here and that significant population size expansion began to occur during the Sui and Tang dynasties ~1500 years ago. We speculate that the reason for the expansion of the ginkgo population is mainly related to the prosperity and development of Buddhism at that time. Buddhism was supported by the government, and Buddhist temples were also funded and protected by the state during the Sui Dynasty, especially in Shandong, which had a high political, economic, and cultural status. Ginkgo has an important position in Buddhist culture. Many monks like to plant ginkgo around the temple and give each other ginkgo seeds. In Korea and Japan, many of the largest or oldest ginkgo trees are associated with Buddhist temples [7]. The Tai’an area has now become the largest ginkgo cultivation area in the world.

Exploring the affinities of cultivars plays an important role in the utilization of germplasm resources, especially in artificial breeding. Most of the ginkgo cultivars are distantly related, except for a few that are closely related due to geographical proximity, which affirms the accuracy of the earlier germplasm collection. The average length of ROHs in ginkgo was small, much smaller than in *Pyrus* spp. [34] and *Saccharum* spp. [35], but the inbreeding coefficients are not very different. The shorter the length of ROHs, the more distant the generation that produces the ROHs. This is also in line with the rule that the generation cycle of ginkgo is greater than that of *Saccharum* spp. In the further analysis of ROH islands, it was found that different subpopulations faced different selection pressures and directions. High compound content is one of the main directions of artificial selection. The improvement of the ability to cope with environmental stress and the enhancement of photosynthesis are the results of natural selection. The highest degree of inbreeding in subpopulation G2 may be related to its concentrated distribution in a small geographical area.

**Table 1:** Candidate genes associated with seed traits of ginkgo.

Trait	SNP	Position	*P*	Genes
Seed length	Chr9_370535479	370535479	5.23361E−09	*Gb_39567*
Seed width	Chr1_357954718	357 954 718	8.68762E−09	*Gb_19888*; *Gb_19887*
	Chr1_491753724	491753724	4.7562E−09	*Gb_07898*
	Chr1_693353185	693353185	1.87639E−09	*Gb_18542*; *Gb_18543*
	Chr1_965826967	965826967	1.00687E−08	*Gb_36948*; *Gb_36946*; *Gb_36945*; *Gb_36944*
	Chr3_278506827	278506827	6.49114E−09	*Gb_00245*
	Chr3_387338727	387338727	6.51533E−11	*Gb_35407*
	Chr3_387338741	387338741	6.51533E−11	*Gb_35407*
	Chr4_165088828	165088828	1.74365E−09	*Gb_20130*
	Chr4_397241238	397241238	9.15228E−09	*Gb_17798*
	Chr5_5688785	5688785	1.40274E−09	*Gb_10777*
	Chr6_36703692	36703692	2.30785E−09	*Gb_29959*
	Chr6_431760453	431760453	1.25951E−10	*Gb_25139*
	Chr6_431836484	431836484	9.70058E−09	*Gb_25139*
	Chr9_301641688	301641688	3.94001E−09	*Gb_20309*
	Chr10_431836891	431836891	1.14319E−09	*Gb_18647*
	Chr11_84427883	84427883	3.24413E−09	*Gb_02867*
	Chr12_345499339	345499339	5.20016E−10	*Gb_33960*; *Gb_33964*
Seed thickness	Chr5_664008421	664008421	1.22487E−08	Gb_20425
Embryo-free rate	Chr11_84427883	84427883	3.24413E−09	*Gb_02867*

Seed traits became the most obvious basis for partitioning different ginkgo cultivars. The phylogenetic topology of candidate genetic regions based on seed shape was basically consistent with that of the whole genome (Supplementary Data File S3), and neither supported the basis for classifying ginkgo seeds based on shape. There was no significant difference between the two topologies, suggesting on the one hand that the SNPs in the GBS data are more evenly distributed, and on the other hand proving that the seed shape cannot fully represent the taxonomy. Since this study represents the first attempt to identify genetic associations with ginkgo seed traits using GWAS, and there have been no prior investigations into regulatory genes for these traits, further validation is needed to confirm the potential candidate genes identified in this analysis. GWAS suggested that loci controlling seed length and seed thickness are mostly independent. Abscisic acid and auxin are key phytohormones involved in the growth and development of fruits and seeds [36]. Based on functional annotation of candidate genes related to ginkgo seed thickness, we can infer that they play a similar role in ginkgo seeds. *CAT* genes exist in multiple copies in plants, and their regulatory patterns and tissue specificity are also different [37]. The structural protein catalase encoded by *CAT* plays an important role in the regulation of ginkgo seed width and embryo-free rate. The content of ginkgotoxin in embryo-free seeds is greatly reduced [38]. Controlling the embryo-free rate of ginkgo seeds by regulating the expression of the *CAT* gene represents a novel approach for enhancing innovation in ginkgo germplasm.

The positioning of biological traits by GWAS has achieved great success, especially providing a cost-effective means for the mapping of long-generation woody trait-associated genes, such as in *Eucalyptus* [39], *Pyrus pyrifolia* Nakai [40], and Korean apple [41], etc. GWAS is not the only way to explore the regulatory loci of biological traits. Seven quantitative trait loci (QTLs) were found to be associated with seed width and two QTLs were associated with seed length in soybean by constructing genetic maps [42]. While constructing hybrid systems through artificially controlled crosses can provide valuable research material for investigating germline regulatory genes, researchers should be aware that the experimental cycles can be exceptionally long, particularly for woody plants with extended generation cycles.

### Conclusion

This study has yielded novel insights into the genetic structure and dynamic history of cultivated populations of ginkgo, as well as the localization of candidate genes that are associated with seed traits. Our large-scale GBS analysis of ginkgo cultivars revealed that they can be grouped into four subpopulations with lower low level genetic diversity, and most of the cultivars displayed no apparent genetic relatedness. The expansion of the G2 and G3 subpopulations can be traced back to 1500 years ago. Through long-term survey and GWAS analysis of seed traits, we successfully located a total of 54 SNPs and 24 candidate genes. Thus, this study improves our understanding of the genetic structure and dynamic history of cultivated ginkgo populations, as well as the candidate genes that are associated with seed traits, which will facilitate the effective conservation and utilization of ginkgo germplasm.

## Materials and methods

### Plant materials

A total of 102 ginkgo cultivars (Supplementary Data File S4) were collected from China (96), Japan (2), and Europe (4). The material from China and Japan was collected in the 1970s and 1980s, and was grafted and preserved in the China Ginkgo Germplasm Resource Base (34.39 N, 118.03 E). Four European germplasm cultivars were added for comparison. We collected fresh leaves of all germplasms, and stored them frozen in liquid nitrogen at −80°C for DNA extraction.

### DNA extraction and genotyping-by-sequencing

DNA extraction from frozen ginkgo leaves was performed using the CTAB method [43]. DNA quality was assessed using Qubit and Nanodrop instruments (Thermo Fisher Scientific, Waltham, MA, USA). Genomic DNA samples that passed the quality check were selected for subsequent GBS library construction. To prepare the samples for library construction, genomic DNA was digested with the restriction enzyme EcoRI-NIaIII and subjected to end repair. The DNA library was prepared using the NEBNext^®^ MLtra™ DNA Library Prep Kit (NEB, USA), with the addition of A-tails and Illumina sequencing adapters. DNA fragments ranging from 300 to 400 bp were PCR-amplified and purified with an AMPure XP system (Beckman Coulter, Brea, CA, USA). Library quality was assessed using an Agilent 2100 Bioanalyzer (Agilent, Santa Clara, CA, USA), and quantification was performed with real-time PCR. Sequencing was carried out using the PE 150 strategy on a Novaseq 6000 sequencer.

FASTP (v.0.18.0) was used to filter the raw data from the Illumina platform [44]. Reads with unknown nucleotides (N) ≥ 10%, phred quality score ≤ 20 with >50% base content and those containing connectors were excluded. The filtered reads were aligned to the latest reference ginkgo genome using the mem algorithm with the software BWA (v.0.7.12) [21, 45]. After alignment, the results were marked using the software picard (v.1.129). Population SNP detection was performed using GATK (v.3.4-46) [46], filtered using VariantFiltration with -Window 4, −filter ‘QD < 4.0 || FS > 60.0 || MQ < 40.0’, -G_filter ‘GQ < 20’. Detected variants were functionally annotated using ANNOVAR (v.2) [47].

All of the raw sequencing data have been submitted to the National Genomics Data Center (NGDC) with the accession number CRA006613, ensuring that the data are publicly accessible and reproducible.

### Population structure analysis

We filtered samples with abnormal heteronomy variants, missing rate of variants >0.2, high heterozygosity for a threshold of mean + 3*standard deviationsd, and those recognized as an outlier were filtered. The ADMIXTURE [48] (v.1.3) program was used for genetic assignment using unlinked SNPs. This unlinked SNP set was selected from filtered SNPs by removing SNPs with LD (*r*^2^) >0.2 using PLINK version 1.90p17 [49]. We ran ADMIXTURE with the cross-validation (CV) flag specifying from *K* = 1 to *K* = 9 clusters, and the one with lowest cross-validation error was chosen as the best *K*. The R package Pophelper [50] (v.2.2.7) was used to generate the ancestry bar plots.

### Principal component analysis, kinship analysis, and linkage disequilibrium analysis

We conducted PCA [51] kinship analysis, and LD analysis to analyze the genetic diversity among the cultivars. To perform PCA, we utilized GCTA (v.1.92.2) software [52] and filtered the SNPs accordingly [52]. To infer the kinship among cultivars, we calculated pairwise genotype probabilities (*P*) of sharing 0, 1, or 2 identity by descent (IBD) alleles at each locus using the method-of-moments algorithm implemented in PLINK version 1.90p17, based on pairwise identity by state (IBS) distances and allele frequencies. LD decays of the whole population and each subpopulation were analyzed using the PopldDecay (v.3.41) software [53] suite based on filtered SNPs.

### Runs of homozygosity detection and island analysis

We utilized Vcftools v.0.1.14 [54, 55] to eliminate indels from the markers. Subsequently, we applied PLINK to filter the SNP sites. This ensured that the resulting dataset contained high-quality SNPs, devoid of any indel markers. At least 100 SNP loci on all chromosomes with a minimum length of 1000 kb of completely homozygous genomic stretches were identified. The distribution of ROH fragments can indicate variations in the number and length of runs of homozygosity (ROH) fragments among different populations, which in turn reflects changes in ROH. We calculated the genome inbreeding coefficient *F*_ROH_ using the ROH method to assess the degree of inbreeding within populations. *F*_ROH_ was calculated by determining the ratio of the total length of ROH fragments in the genome to the total length of the genome. The following formula was used to calculate *F*_ROH_:

$$ {F}_{\mathrm{ROH}}=\sum \frac{L_{\mathrm{ROH}}}{L_{\mathrm{AUTO}}} $$

The ROH ratio for each SNP site was calculated when considering the population as a whole. A Manhattan map was produced based on the ROH ratio of each SNP site, and the threshold line for high-frequency SNPs was set at the ROH ratio where the top 1% of SNPs were located. By examining the distribution of SNP sites exceeding this threshold throughout the genome, ROH islands were identified.

### Acquisition and analysis of phenotypic data

In 2019, 2020, and 2021, phenotype observations were carried out on the seeds of 52 cultivars without the outer testa in the China Ginkgo Germplasm Resource Base. All these cultivars had three clonal repeats and grew in uniform conditions for >40 years. Each phenotypic variable was measured 30 times in each clone, and the tree averages were calculated for each cultivar. The BLUPs of each phenotypic variable were calculated using the Lme4 R package [56] and the normal distribution of the respective BLUP was verified by the Kolmogorov–Smirnov test [57]. BLUP model figures for each phenotypic were generated by the ggplot2 R package [58].

### Genome-wide association mapping

Genome-wide association mapping was implemented in the GCTA (v1.92.2) program [59]; using the mixed linear model accounts for both population structure and kinship as fixed and random effect [MLM(Q + K)].

Population structure was accounted for with the first five principal components (or with the ancestry component matrix corresponding to the best *K* of ADMIXTURE results), whereas kinship was accounted for using the kinship matrix generated by GCTA software. Considering the rare variants and the extremely unbalanced case–control ratios, REGENIE and SAIGE were implemented. REGENIE partitioned SNPs into consecutive blocks and generated ridge regression predictions from each block. Then, a second ridge regression was used to combine the predictors into a single predictor, which was then decomposed into 23 chromosome predictors for GWAS [60]. SAIGE used a saddle point approximation (SPA) [61] to the test statistic null distribution to obtain accurate *P*-values [62]. To improve the power of the association test, we selected variants on the basis of gene region (or other methods for variant set generation) and used GMMAT [63] software for gene/set-based association tests. The framework consisted of three tests: a burden test, a sequence kernel association test (SKAT) [64], and an optimal sequence kernel association test (SKAT-O) [65], all of which utilize the same reduced model with GLM(Q). Current burden tests aggregate variants within a set into a single value, which is then analyzed for association with the trait of interest. SKAT uses a multiple regression model to directly regress the phenotype on genetic variants in a variant set and on covariates, allowing different variants to have varying directions and magnitudes of effects. SKAT-O optimizes the weight of the burden test and SKAT statistics to maximize power, using a grid search over *ρ* to determine the minimum *P*-value.

To determine significant associations, a Bonferroni correction threshold was applied (*P*-value = 0.01/marker number or 0.05/marker number). We then identified candidate genes (CAGs) located within a 50-kb region upstream or downstream of the associated markers.

## Acknowledgements

This research was funded by the National Natural Science Foundation of China (31971648), the China Scholarship Council (202108320301), and the Czech Science Foundation (19-18545S).

## Author Contributions

Y.H. and Q.G. designed the experiments; Y.H., Z.Y., X.G., Y.Z. and G.L. performed experiments; Y.H., Z.Y.,X.G. and P.L. analyzed data; Y.H. and Q.G. wrote the manuscript; P. Š. reviewed the manuscript.

## Data availability

The high-throughput sequencing data in this study are all open access and have been uploaded to the National Genomics Data Center (NGDC, https://ngdc.cncb.ac.cn/) with the accession number CRA006613.

## Conflict of interest

The authors declare no competing interests.

## Supplementary data

Supplementary data is available at *Horticulture Research* online.

## Supplementary Material

Web_Material_uhad136
