# Supplementary material for: Genetic diversity, population structure, and genome-wide association analysis of ginkgo cultivars
Source: Hortic Res. 2023 Jul 11;10(8):uhad136. doi: 10.1093/hr/uhad136 (PMC10410194; doi:10.1093/hr/uhad136)
Supplement: Web_Material_uhad136 [file web_material_uhad136.zip › Supplementary File S1.Variation distribution on 12 chromosomes of ginkgo.docx]

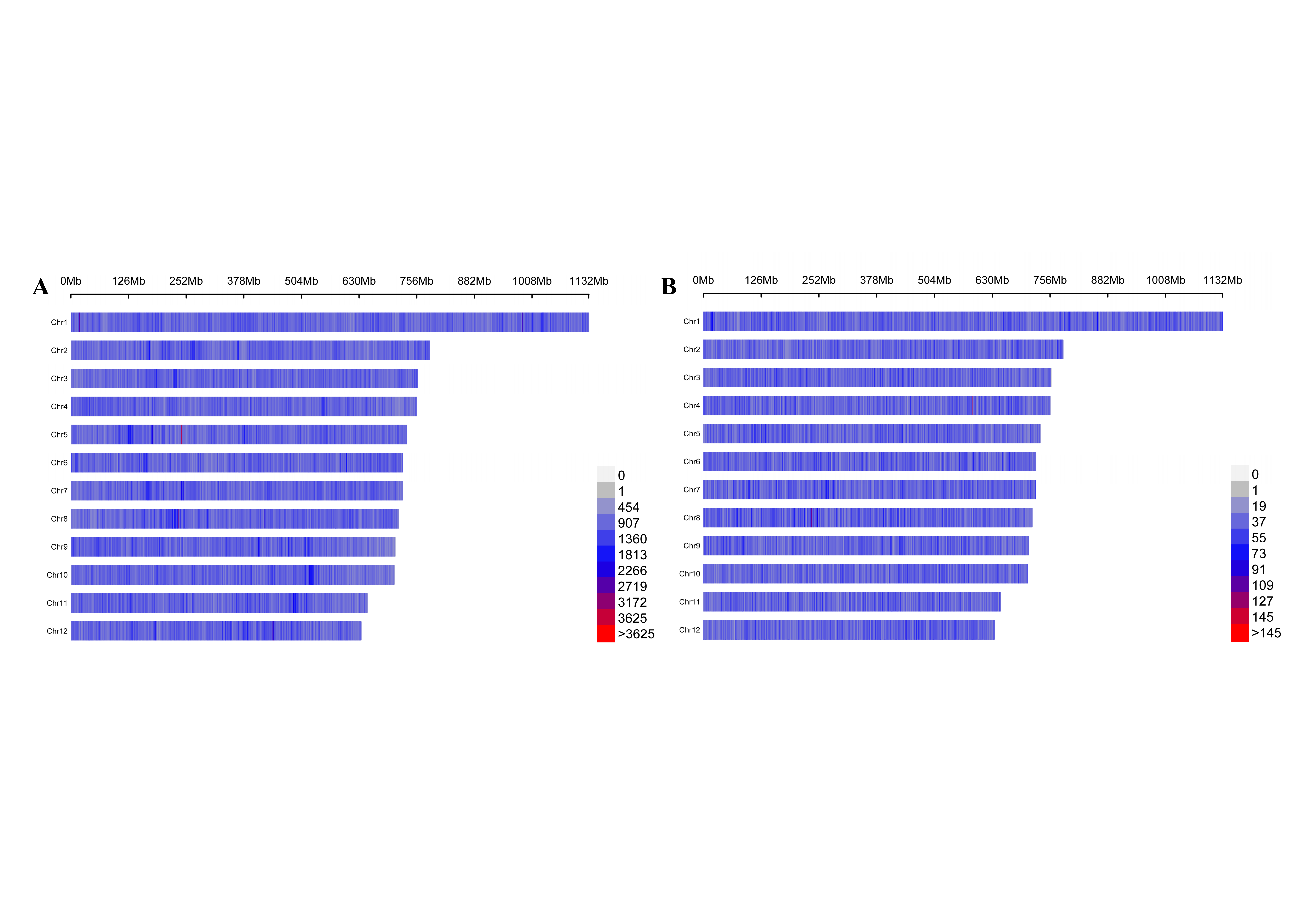


Fig. S1 Variation distribution on 12 chromosomes of ginkgo. A: The distribution of SNPs on chromosomes. B: The distribution of InDels on chromosomes
