# Supplementary material for: Genetic diversity, population structure, and genome-wide association analysis of ginkgo cultivars
Source: Hortic Res. 2023 Jul 11;10(8):uhad136. doi: 10.1093/hr/uhad136 (PMC10410194; doi:10.1093/hr/uhad136)
Supplement: Web_Material_uhad136 [file web_material_uhad136.zip › Supplementary file S2.Frequency distribution of BLUPs.docx]

| 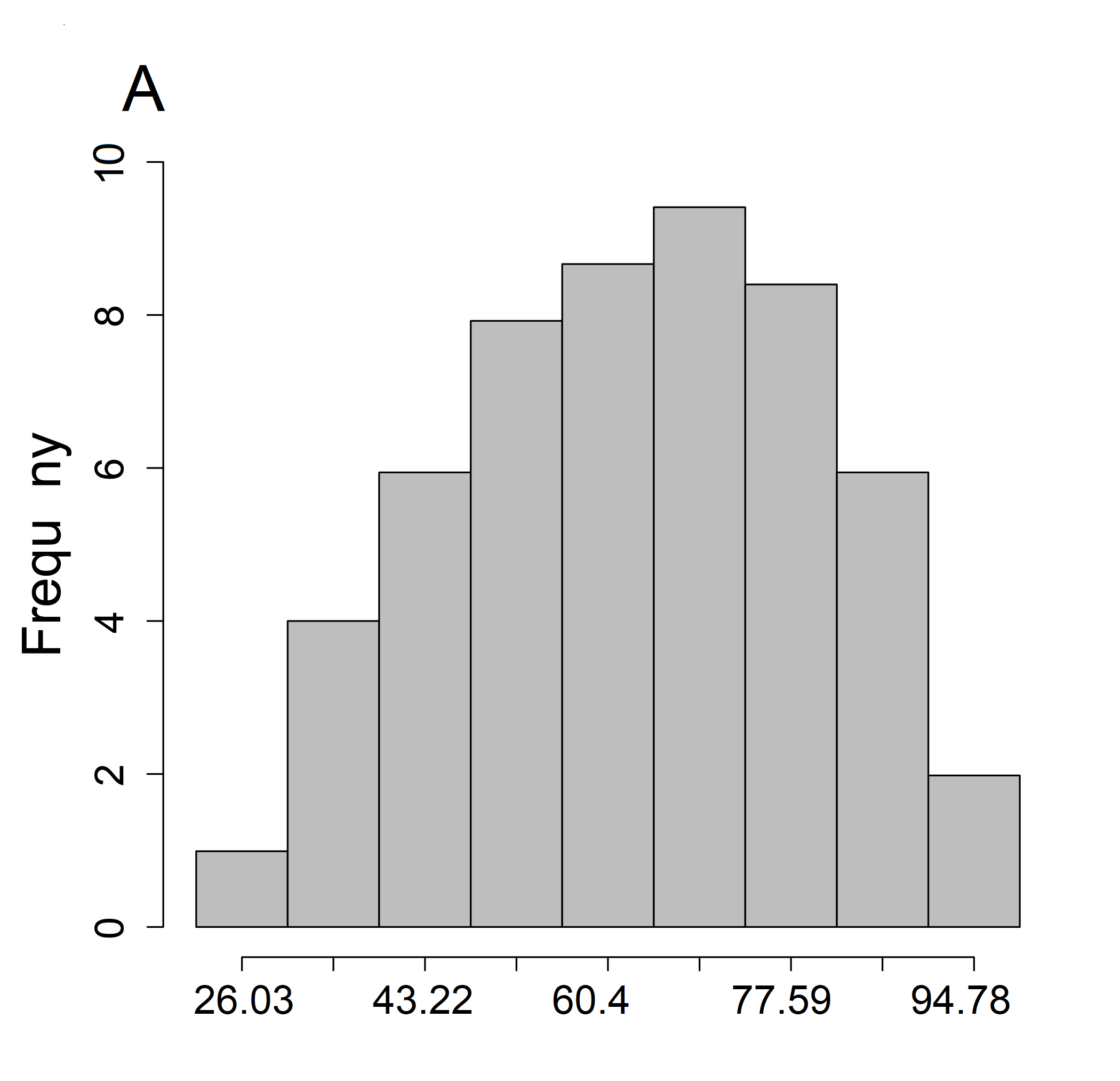 | 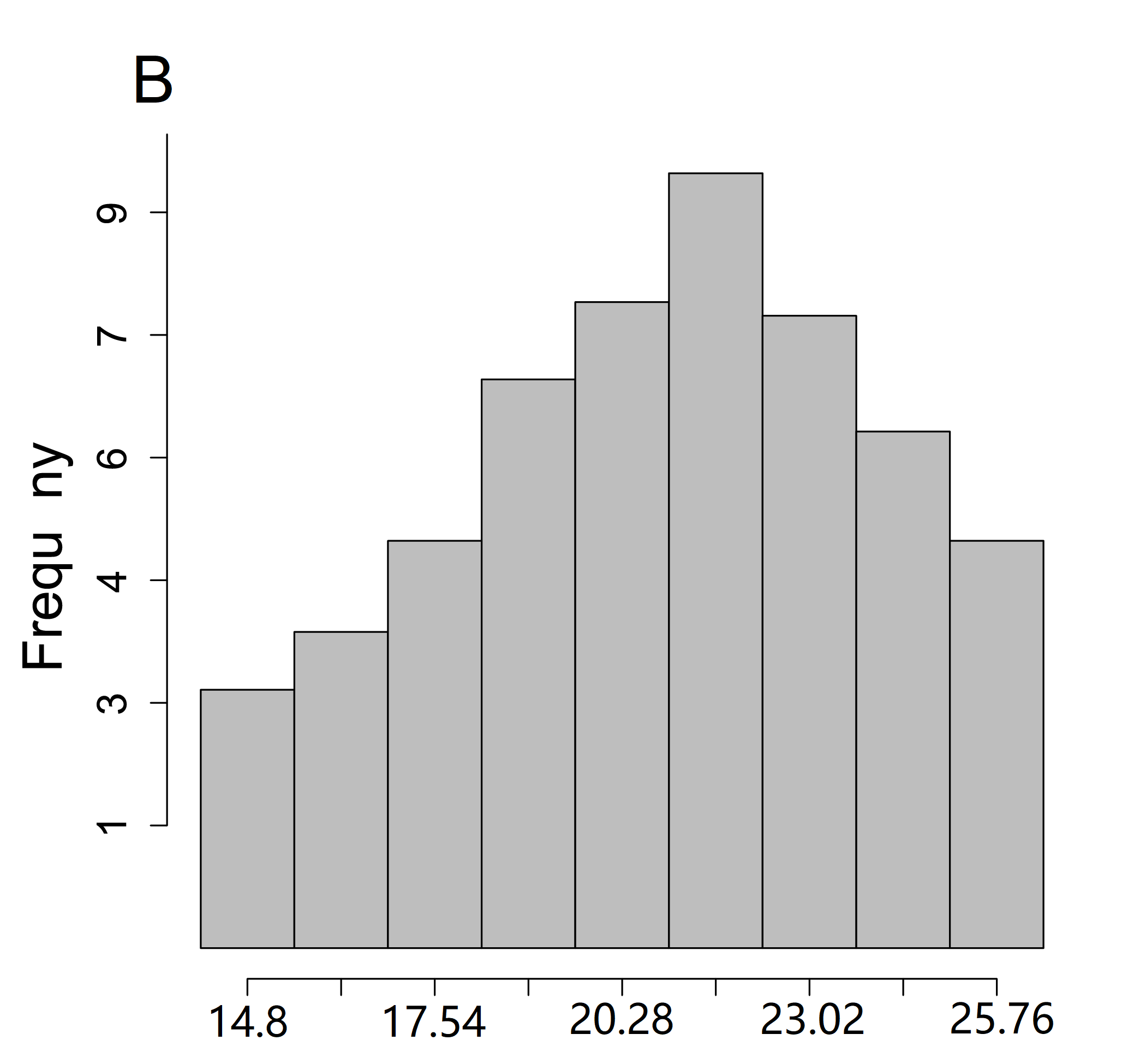 |
| --- | --- |
| 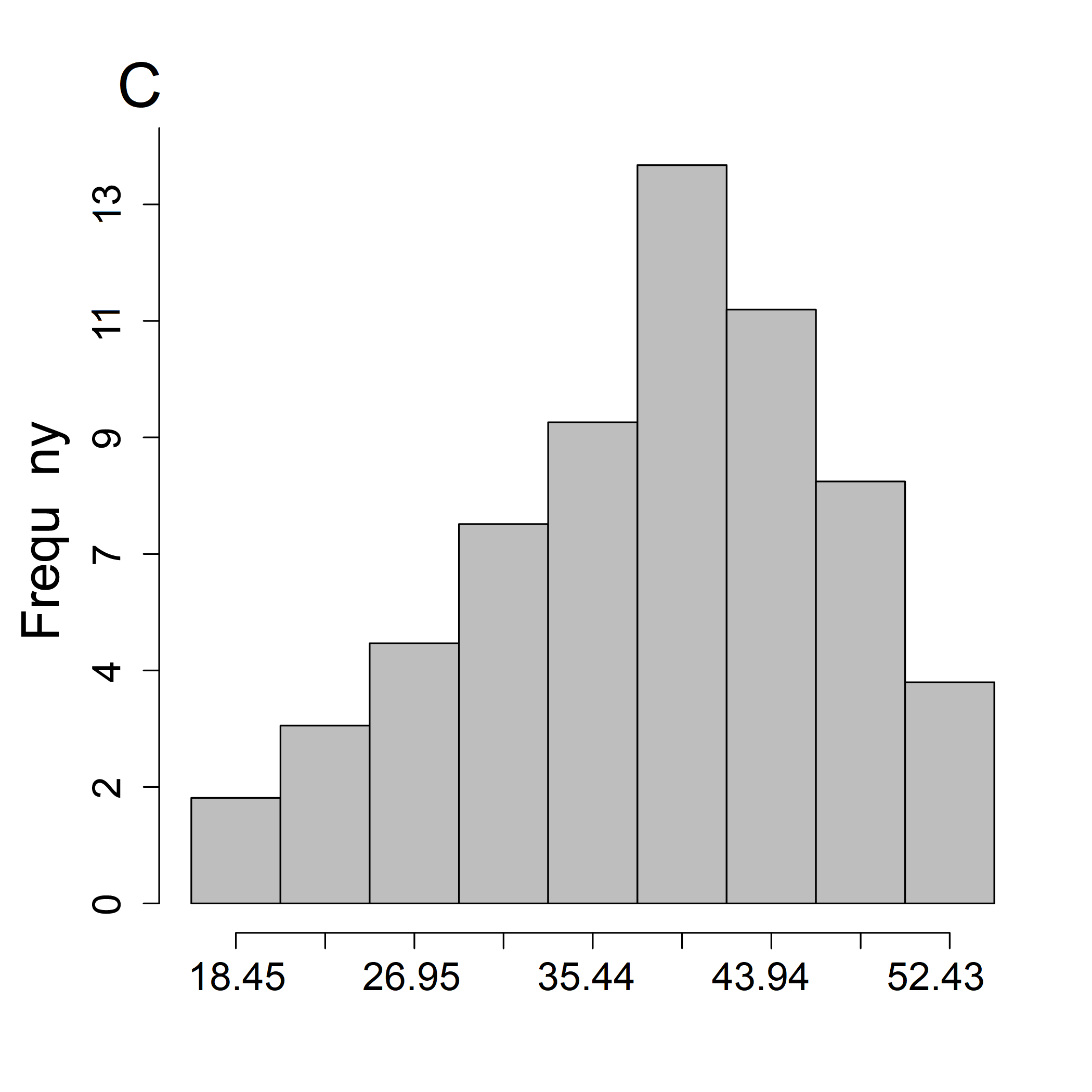 | 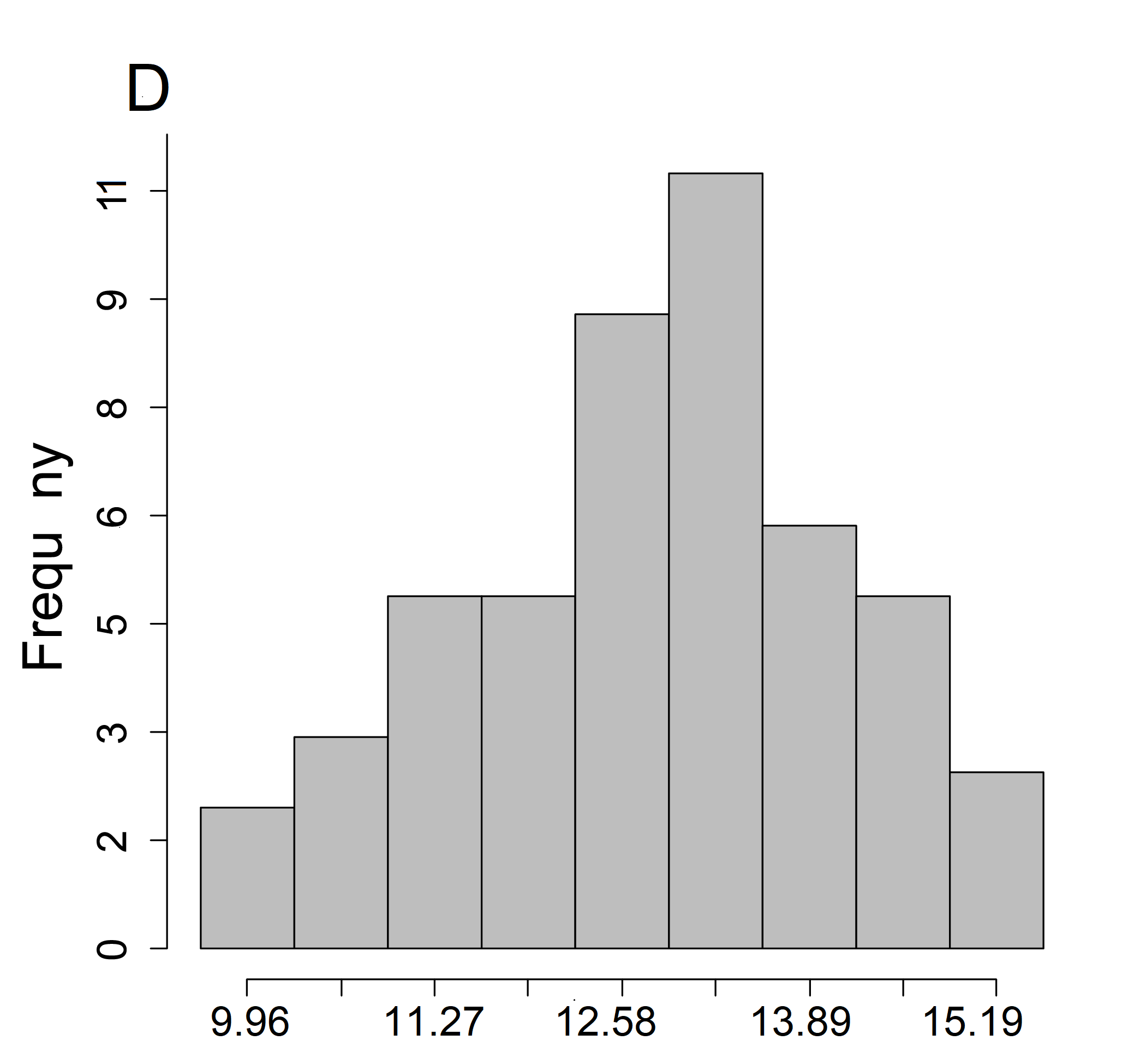 |

**Fig. S2** Frequency distribution of BLUPs for seeds length (A), width (B), thickness (C), and embryo-free rate (D) among cultivars.
