# Supplementary figures and images for: Genetic diversity, population structure, and genome-wide association analysis of ginkgo cultivars
Source: Hortic Res. 2023 Jul 11;10(8):uhad136. doi: 10.1093/hr/uhad136 (PMC10410194; doi:10.1093/hr/uhad136)

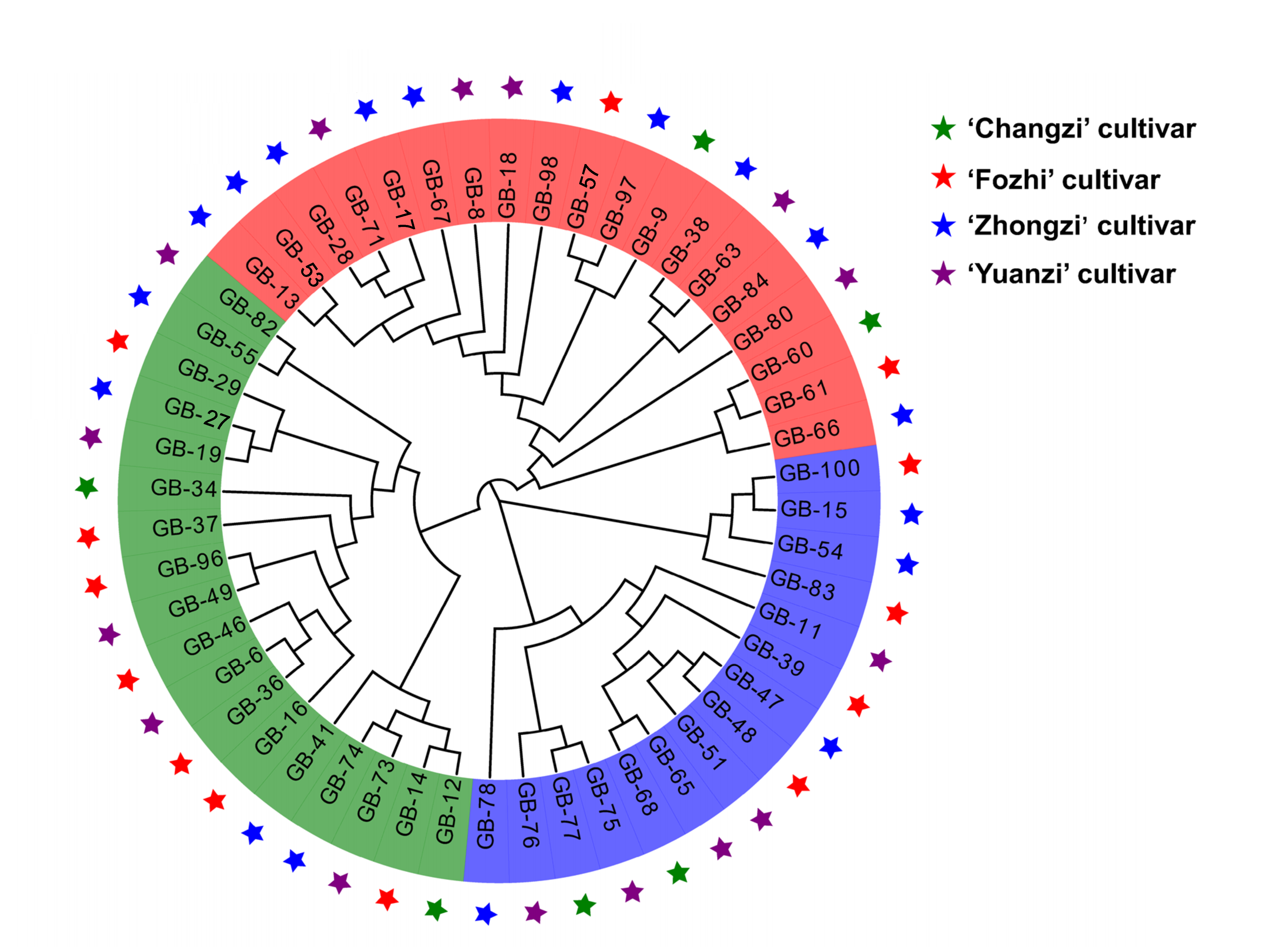


**Fig. S4 Phylogenetic clustering based on whole genomes.**

Supplement: Web_Material_uhad136 [file web_material_uhad136.zip › Supplementary file S3.docx]
